# Mechanisms Underlying Antiarrhythmic Properties of Cardioprotective Agents Impacting Inflammation and Oxidative Stress

**DOI:** 10.3390/ijms23031416

**Published:** 2022-01-26

**Authors:** Katarina Andelova, Barbara Szeiffova Bacova, Matus Sykora, Peter Hlivak, Miroslav Barancik, Narcis Tribulova

**Affiliations:** 1 Centre of Experimental Medicine, Slovak Academy of Sciences, Institute for Heart Research, Dúbravská Cesta 9, 84104 Bratislava, Slovakia; katarina.andelova@savba.sk (K.A.); matus.sykora@savba.sk (M.S.); miroslav.barancik@savba.sk (M.B.); 2 Department of Arrhythmias and Pacing, National Institute of Cardiovascular Diseases, Pod Krásnou Hôrkou 1, 83348 Bratislava, Slovakia; hlivakp@gmail.com

**Keywords:** inflammation, oxidative stress, cardiac arrhythmias, SGLT2i, statins, omega-3

## Abstract

The prevention of cardiac life-threatening ventricular fibrillation and stroke-provoking atrial fibrillation remains a serious global clinical issue, with ongoing need for novel approaches. Numerous experimental and clinical studies suggest that oxidative stress and inflammation are deleterious to cardiovascular health, and can increase heart susceptibility to arrhythmias. It is quite interesting, however, that various cardio-protective compounds with antiarrhythmic properties are potent anti-oxidative and anti-inflammatory agents. These most likely target the pro-arrhythmia primary mechanisms. This review and literature-based analysis presents a realistic view of antiarrhythmic efficacy and the molecular mechanisms of current pharmaceuticals in clinical use. These include the sodium-glucose cotransporter-2 inhibitors used in diabetes treatment, statins in dyslipidemia and naturally protective omega-3 fatty acids. This approach supports the hypothesis that prevention or attenuation of oxidative and inflammatory stress can abolish pro-arrhythmic factors and the development of an arrhythmia substrate. This could prove a powerful tool of reducing cardiac arrhythmia burden.

## 1. Introduction

Cardiac arrhythmias remain a serious global clinical issue. The current treatment of stroke-provoking atrial fibrillation (AF) and life-threatening ventricular fibrillation (VF) employs invasive approaches with implanted cardioversion devices and catheter ablation of pro-arrhythmic triggers [1,2,3,4,5,6,7,8]. Although these approaches can abolish AF and VF, they have no impact on the development and/or recurrence of these arrhythmias. The current antiarrhythmic drugs can also be pro-arrhythmic by overdose or adverse interaction with cardiac disease status [1,9], and this supports the theory that prevention of cardiac arrhythmia development is the best approach in reducing cardiac arrhythmic burden. This has inspired ongoing search for novel approaches which prevent or at least attenuate pro-arrhythmic factors and conditions.

Numerous experimental and clinical studies suggest that oxidative stress [10] and inflammation are deleterious to cardiovascular health [11,12,13] and can increase the heart’s susceptibility to arrhythmias [14,15,16,17,18,19]. While many of the cardio-protective compounds which exhibit antiarrhythmic properties are potent anti-oxidative and anti-inflammatory agents [13,20,21,22,23]. These most likely target the primary mechanisms of pro-arrhythmia. This view concurs with the hypothesis that upstream antiarrhythmic therapy prevents or limits arrhythmia substrates and the pro-arrhythmic factors that promote myocardial electrical instability. Upstream therapy refers to the use of non-antiarrhythmic cardio-protective agents [24,25,26,27,28,29,30,31]. These are able to prevent the occurrence or recurrence of cardiac arrhythmia. The current challenge, however, is to update the guidance for managing cardiac arrhythmias and develop novel and safer antiarrhythmic compounds. The clinical benefits of inflammation-and redox-related therapies [10,32,33] can be achieved by personalized medicine, and this will provide innovative approaches for precision medicine. 

This focused review-article highlights the growing mechanistic link between inflammation and oxidative stress and arrhythmogenesis, and outlines the established and putative mechanisms which underlie the antiarrhythmic properties of selected compounds acknowledged to influence inflammation and oxidative stress. These are the sodium-glucose cotransporter-2 inhibitors (SGLT2i) used in diabetes treatment, the statins for dyslipidemia and the naturally cardio-protective omega-3 fatty acids (Omega-3 FA). Finally, it is emphasized that a healthy lifestyle which avoids cardiovascular risk factors, with their attendant inflammation and oxidative stress, is highly effective in preventing the occurrence of both atrial and ventricular arrhythmias.

## 2. Inflammation and Redox Disorders Linked with Cardiac Arrhythmia Burden

Most cardiovascular pathophysiological conditions involve inflammatory responses and pathways of the immune system captured in the term ‘inflammaging’. Inflammation is an immune response following infections and cellular and tissue demage [34]. Therein, the lipid components of cell membranes and modulators of different biological functions influence the immune responses and inflammatory processes [32,34,35]. These lipids are involved in the pathophysiology of different autoimmune diseases, and excessive pro-inflammatory lipid activity contributes to transition from acute to chronic inflammation.

Inflammasomes are components of the innate immune response, and are involved in tissue homeostasis [32]. The NOD-like receptor protein-3 (NLRP3) inflammasome is involved in the activation of caspase-1 and the maturation of interleukin (IL)-1β and IL-18 [36]. Recent studies implicate connexin (Cx) hemichannels (CxHC) and pannexin-1 channels in the spread of pro-inflammatory purinergic signaling by NLPR3 or NLRC4 inflammasome in the cardiac and vascular systems [17,37]. CxHC’s are activated in pathophysiological settings that promote cardiac arrhythmia development, and NLRP3 inflammasome is a key driver of obesity-induced atrial arrhythmias [38,39]. 

Western diets rich in cholesterol can trigger NLRP3 inflammasome-dependent innate immune re-programming [40]. Gut microbiota dysbiosis which generates bioactive metabolites can exert inflammation-related pro-arrhythmic action [41,42]. In addition, environmental stressors such as air pollutants and artificial night-lighting predispose to the inflammation and oxidative stress-related cardiovascular disorders that promote arrhythmias [43,44]. It is, therefore, hypothesized that CxHC inhibition is a worthy target for inflammation treatment and arrhythmia prevention [17].

The important recently discovered inflammatory and non-canonical functions of cardiac immune cells indicate that leukocytes can be arrhythmogenic by either altering tissue composition or interacting with cardiomyocytes [45]. While cardiac inflammation can be enhanced by sympathetic overdrive [46], the β1-adrenergic receptor blockers abrogate inflammation [47]. In addition, some authors consider that metabolic inflammation is central to the pathophysiology of heart failure (HF), with preserved ejection fraction [48], while NLRP3 inflammasome activation contributes to chronic inflammation in HF with reduced ejection fraction [37]. Most recent study provides evidence for genetically driven systemic inflammation in cardiovascular diseases, and it highlights the NLRP3 inflammasome as a therapeutic target [49]. The inflammation process involves numerous cytokines produced by different molecular pathways with multiple functions [7,18,50], and it is further associated with the over-abundant reactive oxygen species (ROS) mostly produced by mitochondria [51,52]. This can trigger NLRP3 inflammasome activation [53,54]. 

The mitochondria are energy producers which generate ATP through oxidative phosphorylation driven by electron transport across the electron transport chain. They concurrently generate ROS which have diverse cell signaling function [25,52,55]. Cardiovascular diseases accompanied by disrupted mitochondrial membrane potential alter both cardiac myocyte energy production and redox balance. Moreover, mitochondrial dysfunction is a hallmark of most cardiovascular disorders [10,33] and promotes cardiac electrophysiological abnormalities at the cellular level and subsequent malignant arrhythmias [55]. Oxidative stress affects protein translation and contributes to heart propensity to arrhythmias and cardiovascular disease [56]. Therefore, accurate evaluation of redox status and precise application of antioxidants could be an efficient therapy of suppressing oxidative stress and the development of cardiac arrhythmia [10,25,33].

New strategies targeting redox regulation and inflammation are needed to eliminate combined pro-arrhythmic risk factors [15,57,58]. Recent clinical trials have revealed that corticosteroids and colchicine may prevent AF development and support the fundamental impact of inflammatory pathways in managing AF [7]. Colchicine taken up by leucocytes inhibits cytokine and interleukin expression and modulates leucocyte superoxide production [59]. However, there is still limited data on the therapeutic implications and clinical benefit of primary anti-inflammatory agents in arrhythmia management. 

The impact of acute and/or chronic inflammation and oxidative stress in stroke provoking AF and life-threatening VF is suggested by numerous experimental and clinical studies [3,4,6,7,15,41,53,55,60,61,62,63,64,65,66,67,68]. The cardiovascular risk factors that predispose to arrhythmias are accompanied by low grade inflammation and oxidative stress. These factors include hypertension, obesity, insulin resistance, metabolic syndrome, ageing, neurological disorders and circadian rhythm disruption [16,38,40,63,69,70,71,72,73,74,75,76,77,78,79,80]. These conditions cause pro-arrhythmic load and predisposition to acute heart attack and stroke. While the NFκB proinflammatory transcription factor is a key component in crosstalk between inflammation and cardiovascular disease [81]. The ultimate result, however, can be inflammatory aggravation and oxidative stress. 

Redox status disorders and increased pro-inflammatory cytokines lead to cardiac channelopaties. These are dysfunctions of ion channels as well as Cx43 and Cx40 gap junction channels and they include CxHC activation [7,17,82,83,84,85] and the abnormal Ca^2+^ handling that underlie myocardial electrical disorders [7,8,27,55,65,66,68,86,87,88,89,90]. Ca^2+^ channels, including the sarcoplasmic reticulum Ca^2+^ release RyR channels and transporters, are under redox regulation [88,91], and abnormal Ca^2+^ handling is fundamental in the induction and maintenance of AF and VF [66,91,92]. 

**Figure 1:**
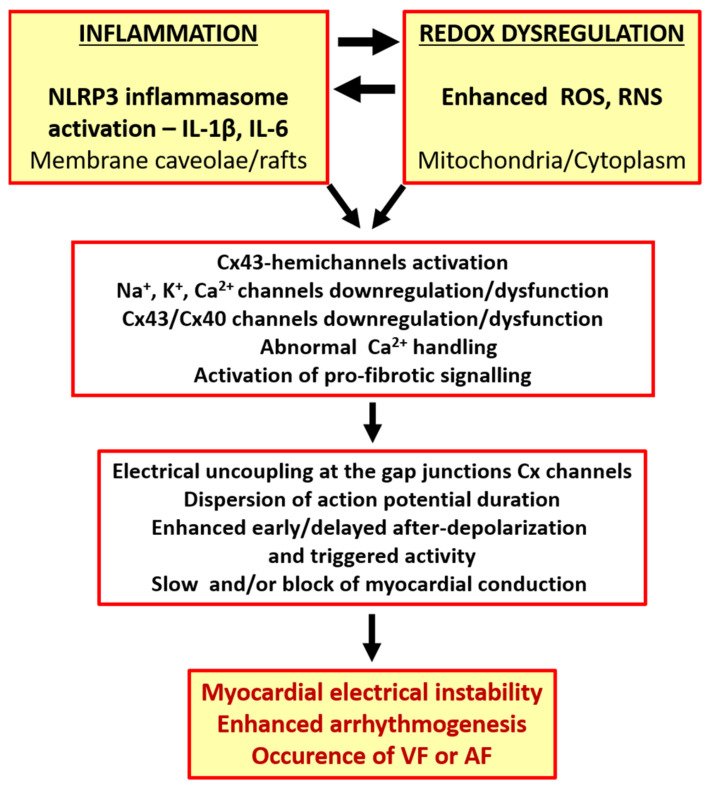
The link between the occurrence of cardiac arrhythmias and inflammation or redox dysregulation.

In addition, down-regulation of the Cx43 or Cx40, alterations in their distribution, and gap junction channel dysfunction and uncoupling can all unmask ectopic foci, increase action potential duration dispersion and refractoriness and slow myocardial conduction [93,94,95,96,97,98,99,100,101,102,103]. These conditions promote the malignant arrhythmia triggers of early or delayed after-depolarizations and the slowed conduction and/or conduction block that facilitate AF and VF [2,4,5,91,98,101,104,105,106]. While inflammation and oxidative stress activate CxHC mediated inflammasome signaling, they inhibit Cx43 gap junction channel-mediated electrical signal propagation between cardiomyocytes [17]. This results in electrical remodeling and instability [104], and it contributes to increased arrhythmogenesis and the heart propensity for arrhythmias (as depicted in Figure 1).

The enhanced pro-inflammatory and ROS signaling facilitate AF and VF occurrence [4,25,50,55,68,105,107], and it is noteworthy that AF results in the myocardial mechanical dysfunction that promotes atrial thrombus formation and stroke, and VF increases the risk of sudden cardiac death (SCD).

Combined inflammation and oxidative stress are causal factors in arrhythmogenesis, and attacking these early pro-arrhythmic factors and their down-stream targets could help improve the management of cardiac arrhythmias and cardiovascular outcomes in high risk individuals and patients with cardiovascular disease. This concurs with evidence that supports the antiarrhythmic effectiveness of non-antiarrhythmic agents with anti-inflammatory and antioxidant efficacy. The following chapters herein present updated findings on the antiarrhythmic properties and the established and putative underlying mechanisms of: (1) the sodium-glucose transport protein 2 inhibitor (SGLT2i) novel drug for diabetes treatment; (2) the statins, and especially atorvastatin, which are the most widespread long-standing treatment for dyslipidemia and (3) natural cardio-protective omega-3 FA used in numerous clinical trials.

## 3. Antiarrhythmic Efficacy of SGLT2 Inhibitors

The SGLT2is increase glucose excretion by blocking kidney reabsorption. Therefore, SGLT2i such as empagliflozin are efficacious in patients with type 2 diabetes (T2DM) [108]. T2DM is accompanied by low-grade inflammation and oxidative stress and independently associated with AF [109]. T2DM forms part of the metabolic syndrome cluster which includes obesity and hypertension. Of note, body weight loss following SGLT2i treatment has been associated with a lower risk of new-onset AF in patients with T2DM [110]. In addition, meta-analysis suggests that epicardial fat is significantly reduced in T2DM patients with SGLT2-i treatment [111]. While epicardial fat is known to promote AF or VF due to the activation of inflammasome and production of cytokines [38,39,112]. Advanced glycation end-products and activation of their receptors may also confer a signaling mechanism for diabetes-related AF [113]. The EMPA-REG OUTCOME trial recorded that patients with T2DM and AF may especially benefit from the use of empagliflozin [114] and SGLT2i were associated with a lower risk of new-onset AF in T2DM patients compared to the dipeptidyl peptidase-4 inhibitor [115]. In addition, a large pharmaco-vigilance database highlighted that AF occurred more frequently in diabetes when medications other than SGLT2i were used [116]. Therefore, SGLT2i may confer a specific AF or atrial flutter-reduction benefit not only in T2DM patients but also in high risk populations [117,118]. The treatment with SGLT2i also significantly reduced the development of new-onset AF in the T2DM patients who had non-ischemic dilated cardiomyopathy [119].

In addition, SGLT2i treatment was associated with the reduction of new-onset HF and cardiovascular mortality [120]. The cardioprotective effect of SGLT2i was independent of glycemic control, diabetes or a reduction in traditional cardiovascular risk factors. Moreover, the DECLARE-TIMI 58 trial showed a 19% AF reduction in DM patients with SGLTi treatment, regardless of pre-existing AF or HF [121]. These findings complement available evidence from trials supporting protective SGLT2i pleiotropic effect against the occurrence of AF. SGLT2i also reduced the risk of cardiac arrhythmias in patients with DM, HF and chronic kidney disease [122,123].

Moreover, dapagliflozin SGLT2i reduced the risk of serious VT or VF, cardiac arrest or SCD when combined with conventional therapy in patients with HF with reduced ejection fraction [124]. However, further research is still needed to determine overall risk of SCD and ventricular arrhythmias in patients with T2DM and/or HF treated with SGLT2i [125]. Suppressed ventricular ectopic burden after 2-weeks treatment with dapagliflozin suggests early antiarrhythmic benefit in patients suffering from HF with reduced ejection fraction [126]. There is also direct evidence decoding the effects of SGLT2i on ventricular arrhythmias in HF [127,128,129], and also reversion of cardiac remodeling and improved cardiac function [130,131]. Nevertheless, future studies are required to elucidate mechanistically cardiovascular benefit of SGLT2i for more specific targeting of HF therapy [20]. 

Single empagliflozin dose was well tolerated by healthy volunteers, and it was not associated with QTc prolongation [132]. Ongoing experimental studies suggest that SGLTi not only attenuates HF but also counteracts cellular ROS production in cardiomyocytes, thereby potentially hampering myocardial remodeling and reducing AF and VF burden. This important feature of SGLT2i is linked to their impact on redox signaling in AF, and this has recently been comprehensively reviewed [133]. SGLT2i also exerts direct anti-inflammatory and anti-oxidative effects on resting endothelial cells, and its anti-oxidative effect could be partly mediated by NADPH oxidase inhibition [134]. Moreover, chronic SGLTi treatment has been shown to protect diabetic mice from inflammation [135]. These cardioprotective effects appear to be associated with the increased ketone bodies which are acknowledged modulators of inflammation by inhibiting NLPR3 inflammasome and oxidative stress through protective mitochondria function [136]. It is note-worthy that SGLT2i reduced the inflammation and ameliorated clinical outcomes at the five-year follow-up of T2DM patients who had coronary artery bypass grafts [137].

These clinical and experimental studies further suggest that SGLT2i’s have anti-inflammatory and anti-oxidative potential, and that they reduce both blood glucose and cardiac arrhythmia risks. It is therefore highly likely that prevention and/or attenuation of myocardial inflammation and oxidative stress results in the suppression of pro-arrhythmogenic factors due to SGLT2i preservation of ion channels and Ca^2+^ handling. Finally, cardiologists would really appreciate a practical guide that ensures greater understanding of when, how and to whom SGLT2i should be prescribed.

### Mechanisms Relevant to Antiarrhythmic Properties of SGLT2 Inhibitors

Post-ischemic empagliflozin treatment in animal studies was associated with decreased VF induction [65]. This antiarrhythmic effect was linked with improved myocardial redox state and cytosolic Ca^2+^ dynamics. The modification of diastolic Ca^2+^ and cardiac alternants may be responsible for the reduced ventricular arrhythmia. There was also improved mitochondrial respiratory chain function. This respiration complex II contributes to empagliflozin’s post-ischemic cardio-protection from infarction [138], and empagliflozin pre-treatment protects the heart from lethal ventricular arrhythmia induced by myocardial ischemia and reperfusion injury. These protective benefits may occur as a consequence of activation of the ERK1/2-dependent cell-survival signaling pathway in a glucose-independent manner [139]. It is further reported that dapagliflozin attenuated vulnerability to arrhythmia due to ROS suppression and Cx43 up-regulation through the AMP-activated protein kinase pathway in post-infarct rat hearts [140]. Canagliflozin suppressed myocardial NADPH oxidase activity and improved NOS coupling via SGLT1/AMPK/Rac1 signalling, leading to global anti-inflammatory and anti-apoptotic effects in the human myocardium [21].

Blood glucose concentrations greater than 270 mg/dL (15 mmol/L) lead to QT/QTc prolongation and also reduction in potassium IKr [141]. While dapagliflozin suppressed prolonged ventricular-repolarization in insulin-resistant metabolic syndrome rat models [142]. Acute dapagliflozin administered to rats prior to cardiac ischemia had cardio-protective effects by attenuating infarct size, increasing ventricular function, reducing the arrhythmia score and prolonged time to VT/VF onset [143]. Dapagliflozin also suppressed cardiac fibrosis and endoplasmic reticulum stress and improved hemodynamics in the HF rat model [144]. In addition, empagliflozin prevented sotalol-induced QT prolongation. This was most likely achieved by regulating the intracellular Na^+^ and Ca^2+^ balance, and possibly promoting potassium (IKr) channel activation [145]. Moreover, empagliflozin has been shown to modulate Ca^2+^ handling by lowering cytosolic Na^+^ and Ca^2+^ through inhibition of the Na^+^/H^+^ (NHE1) exchanger and the Ca^2+^ L-type channel and SERCA2a, combined with modulation of electrophysiological APD and QT interval shortening [146,147,148]. The salutary effects of SGLT2i on Na^+^ homeostasis by influencing NHE1 activity, and the late INa and calcium/calmodulin-dependent kinase II (CaMKII) activity have been comprehensively discussed [149].

Empagliflozin significantly shortened the QT, attenuated the down-regulation of myocardial Cx43 expression and reduced fibrotic areas in the ventricles of mice with metabolic syndrome [150]. The combined sotagliflozin SGLT1-2i ameliorated atrial remodeling in a rat model with metabolic syndrome-related to HF with preserved ejection fraction [151], and it also suppressed Ca^2+^-mediated in-vitro cellular arrhythmogenesis. This included the magnitude of spontaneous arrhythmic Ca^2+^ release, mitochondrial Ca^2+^ buffer capacity and diastolic Ca^2+^ accumulation. This was most likely achieved by increased Na^+^/Ca^2+^ exchanger forward-mode activity. The over-expression and Ca^2+^-dependent activation of CaMKII are hallmarks of HF. This leads to contractile dysfunction and arrhythmias. In addition, empagliflozin reduced CaMKII activity, and the CaMKII-dependent SR Ca^2+^ leak and improved Ca^2+^ transients may contribute to antiarrhythmic and contractile functions which enhance the empagliflozin effect in HF [152].

Atrial structural and electrical remodeling also facilitate AF development. Canagliflozin suppressed oxidative stress and interstitial fibrosis with improved effective refractory period and conduction velocity in the rapid-pacing dog model [153]. In addition, mitochondrial dysfunction drives structural, electrical and myocardial tissue contractile remodeling in pathophysiological settings. Here, the empagliflozin ameliorated inflammatory burden and atrial fibrosis in T2DM rats, and also improved their mitochondrial function and reduced inducible AF [154]. Empagliflozin has been reported to maintain mitochondria related cellular energetics and afford its benefits against developing adverse remodelling in post-infarction mice [23].

**Figure 2:**
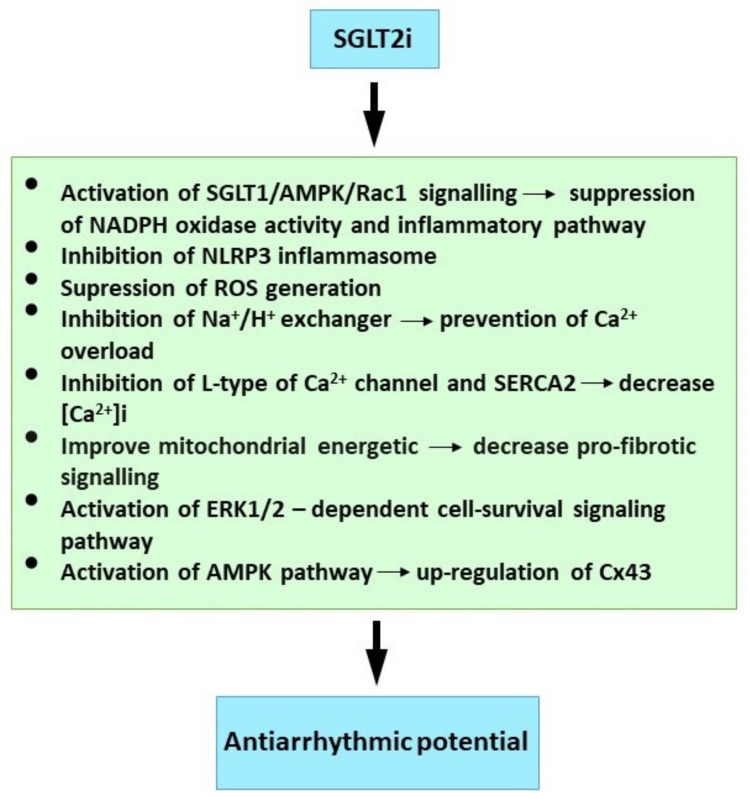
Molecular mechanism of SGLT2i underlying their antiarrhythmic properties. See references [20,21,23,139,140,146,147,148].

Available findings imply that SGLTi may affect Ca^2+^ handling, Na^+^ balance and mitochondrial ROS release. However, further research is required to elucidate SGLT2i’s protective mechanism against cardiac arrhythmias. Finally, although evidence from clinical trials and experimental studies on the molecular mechanisms is compelling (Figure 2), early trials designed specifically for SGLT2i antiarrhythmic efficacy will be welcome, and these should prove informative.

## 4. Antiarrhythmic Efficacy of Statins

Statins as 3-hydroxy-3-methylglutaryl-coenzyme A reductase inhibitors are current first-line therapy in dyslipidemia disorders. Statins lower undesirable lipid-levels and thereby reduce cardiovascular mortality. Statins also exert anti-inflammatory, anti-ischemic, antioxidant and autonomic nervous system modulation [155,156] and they have been shown to suppress toll-like receptors and cytokine expression both in vitro and in vivo. For example, simvastatin reduced circulating TNF-α and MCP-1 in healthy male volunteers [157], and atorvastatin reduced the TNF-α, IL-1b, and IL-6 levels in hypercholesterolemic patients [158]. It is also important that statins suppressed the NLRP-3 inflammasome pathway [159] and up-regulated the cytokine signaling-3 plasma suppressor [160]. Statin therapy was associated with suppression of microbiota dysbiosis [161], which generates pro-inflammatory metabolites promoting arrhythmias [42].

In addition, the statins reduce VT, VF and SCD incidence and AF occurrence. This is attributed to their pleiotropic effects [155]. Statin use in adjunct therapy lowered mortality in patients with HF originating from any cause, and also those with VT [162,163,164] and SCD due to malignant VT/VF [164,165,166,167]. Statins are associated with significant reduction in VT in cardiomyopathy patients with implanted cardioverter defibrillators [168] and myocardial infarction [169,170], and they also reduced postoperative cardiac arrhythmia in patients undergoing arthroplasty [171,172]. Authors further reported that atorvastatin treatment; (1) improved heart rate variability in persons with sleep deprivation [173]; (2) reduced occurrence of exercise-induced premature ventricular contractions [155] and (3) decreased occurrence of post-cardiac surgery-associated AF (POAF) [174,175,176]. However, further studies are required to find the most effective statin regimen for POAF prevention with the highest health benefits [177]. Statin therapy prevented post-reperfusion atrial nitroso-redox imbalance in patients on pump-cardiac surgery [22].

The combination of atrial remodeling and ROS suppression may explain why statins are effective in primary AF prevention [178]. For example, the P-wave dispersion use for AF prediction was lower in cryptogenic stroke patients previously treated with statins [179], and this dispersion correlated with highly-sensitive C-reactive protein (hs-CRP)levels. These reflect inflammation’s role in promoting the slowed and inhomogeneous atrial conduction. Short-term intensive statin therapy reduced the volume of epicardial adipose tissue which is recognized as a pro-inflammatory marker in AF patients [112].

Rosuvastatin reduced autonomic nerve-sprouting combined with decreased mRNA and tyrosine hydroxylase protein expression levels in atrial tissues following acute myocardial infarction [180]. These findings provide understanding of the mechanism statins use to decrease the risk of AF occurrence after heart attack.

Statin use was also associated with lower AF recurrence rate after treatment using catheter ablation of the triggers [181]. A future systematic review and network meta-analysis on statin effects in preventing AF recurrence should provide comprehensive evidence-based proof in clinical practice [182]. Statins could therefore be a novel strategy to prevent AF occurrence in patients with pacemakers, and especially those with sinus node dysfunction [183]. The AF risk associated with premature atrial complexes in patients with hypertension could potentially be reduced by treatment with statins [184]. In addition, a nation-wide study has established that statins reduced new-onset AF after acute myocardial infarction [185].

Statins also attenuated circadian variation in QTc dispersion, and they reduced this pro-arrhythmic parameter in diabetic patients and those who had myocardial infarction [155,186,187]. Statins were also suggested as an adjuvant therapy in reducing AF and VF burden in various clinical settings [188,189], and preoperative statin treatment was shown to reduce VF development and decrease C-reactive protein levels in post-surgery patients [190].

The statin antiarrhythmic potential has been demonstrated in experimental studies. Acute administration of atorvastatin reduced rat heart susceptibility to VF, and long-term atorvastatin treatment was efficacious in rats suffering hereditary hypertriglyceridemia [191,192]. In addition, spontaneous VF during ischemia-reperfusion was suppressed by rosuvastatin [193]. Moreover, the following atorvastatin effects have also been noted; (1) atorvastatin protected the rat myocardium from ischemia-reperfusion induced arrhythmias [194,195]; (2) it ameliorated rat cardiac sympathetic nerve remodeling and prevented VT/VF following myocardial infarction, and it down-regulated IL-1β and TNF-α expression [196]; (3) its antiarrythmia effects may also be due to IL-1β and IL-6 suppression in ouabain-induced rat arrhythmias [197] and (4) it reduced elevated hs-CRP, IL-6 and TNF-α which correlate with longer effective refractory period in the sterile-goat pericarditis model [198]. In contrast, non-treated peri carditis animals had a longer AF duration than the treated group. Anti-inflammatory and anti-ischemic effects form the likely mechanism for the reduced SCD with statin use [155].

### Mechanims Relevant to Statins Antiarrhythmic Properties

Although it has been hypothesized that statins alter the cardiac cell lipid membrane for transmembrane ion channel penetration, the precise statinVT/VF and SCD reduction mechanism has not been established. The penetration may occur by statin-induced modification of lipid rafts which are membrane micro-domains containing signaling molecules and ion channel regulatory proteins [199,200,201]. Thus, statins may directly reduce arrhythmias by favorably altering ion channel conductance. Statins have been shown to reduce hypercholesterolemia and ischemia-reperfusion-induced electrophysiological remodeling in animal models [169,202], and statin’s pleiotropic effects may be due to the inhibition of isoprenoid intermediates [203], because isoprenoid inhibits the Rac and Rho GTP binding proteins. Targeting down-stream Rho kinase could be a predominant mechanism in statin antiarrhythmic effects.

Atorvastatin blocks increased L-type Ca^2+^ current and myocardial cell injury induced by angiotensin II through the inhibition of ROS mediated Nox2/gp91^phox^ and p47^phox^ [204]. It was demonstrated that diabetic rat atrial myocytes had significantly reduced L-type Ca^2+^ current, but increased T-type which was reversed by rosuvastatin [205]. This statin attenuated or reversed SERCA2a, phosphorylated Cx43 and phospholamban down-regulation in the ischemia/reperfusion. Rosuvastatin also accelerated Cai decay and ameliorated conduction inhomogeneity. These are abnormal in injured myocardium. Statins also reduced the ryanodine receptor-2 (RyR2) cardiac activity [206,207], and this suppressed cardiac arrhythmias. In addition, simvastin acetylcholine activated K^+^ current attenuation and shortened APD restoration in mouse atrial cardiomyocytes [208] could be one AF prevention mechanism.

Atorvastatin up-regulated the myocardial Cx43 protein and also activated the phosphatidylinositol-3-kinase pathway and mitochondrial ATP-sensitive K^+^ channels in the ischemia-reperfusion rat model [194]. The myocardial Cx43 up-regulation was also demonstrated in the hereditary hyper-triglyceridemia rat strain [191]. These studies indicate that Cx43 preservation may be implicated in VF protection by statins.

Further statin reports included; (1) pravastatin decreased the incidence of post-myocardial infarction VT and Ca^2+^ alternans in mouse hearts. This was partly achieved by reversing Ca^2+^ handling abnormalities through the protein phosphatase pathway [209]; (2) simvastatin administered prior to ischaemia/reperfusion reduced VF incidence, and treatment preserved endothelial nitric oxide synthase activity and NO availability during occlusion, and attenuated superoxide production following reperfusion [210]; (3) PI3-kinase/Akt pathway activation was involved in acute simvastatin effects against ischemia/reperfusion-induced arrhythmias in anaesthetized dogs [211]; (4) atorvastatin also normalized the myocardial expression level of miRNA-1 known to be involved in Cx43 regulation in rats exposed to irradiation [212]; (5) atorvasatin inhibited the transient Na^+^ current (I_Na_) abnormally increased in rat cardiomyocyets in early ischaemia or reperfusion [213,214] and (6) endothelial Klf2-TGFβ1 or Klf2-Foxp1-TGFβ1 pathway-mediated preventive effects of simvastin against pressure overload induced maladaptive cardiac remodeling [215].

The suppressive effect of rosuvastatin on atrial tachypacing-induced cellular remodeling was mediated by the activation of Akt/Nrf2/HO-1 signaling. This is a possible explanation for the statin AF protective effect [216]. It is also noteworthy that heme oxygenase-1 is a potent antioxidant factor, and the suppression of atrial myeloperoxidase and MMP-2, MMP-9 may contribute to the prevention of atorvastatin in atrial remodeling in the rabbit model of rapid pacing-induced AF [217].

**Figure 3:**
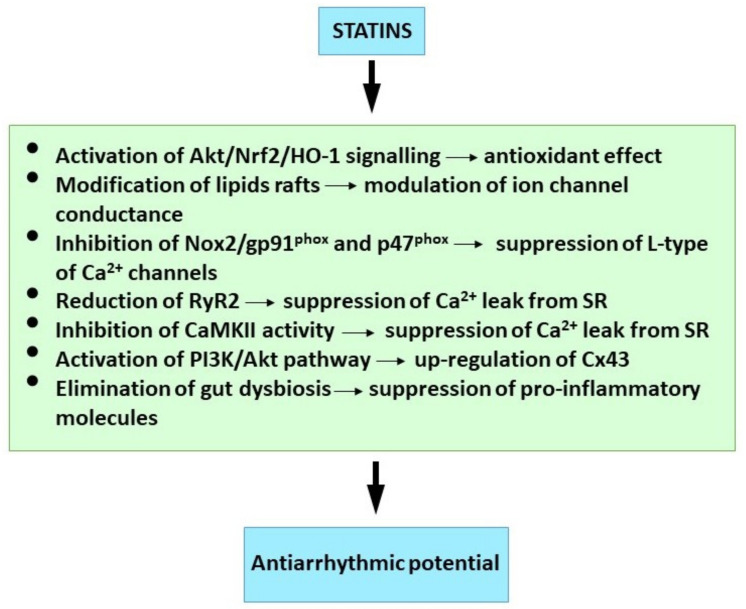
Molecular mechanism of statins underlying their antiarrhythmic properties. See references [42,152,161,194,201,204,206,216].

Available findings suggest that attenuation of inflammation and oxidative stress as well as modulation of ion channels, Ca^2+^ handling, Cx43 and specific signalling pathways may be implicated in antiarrhythmic effects of statins (Figure 3).

## 5. Antiarrhythmic Efficacy of Omega-3 Fatty Acids

It has been accepted for more than 50 years that omega-3 fatty acids exhibit cardio-protective effects [218]. The anti-inflammatory action of these omega-3 FA is mediated by the replacement of arachidonic acid in cellular membranes [219]. Instead the formation of pro-inflammatory mediators like prostaglandins or thromboxanes from the arachidonic acid (AA), the release of eicosapentaenoic acid (EPA) and docosahexaenoic acid (DHA) results in formation of anti-inflammatory mediators like resolvins and protectins. These inhibit pro-inflammatory cytokines [220]. In addition to omega-3 FA’s anti-inflammatory effect, its double bonds are prone to oxidation, so it subsequently activates the Nrf2-pathway promoting endogenous anti-oxidative mechanisms [221]. 

The anti-oxidative benefits and anti-inflammatory or antiarrhythmic effects of several natural compounds have recently emerged in preclinical studies [222,223]. Despite omega-3 FA’s potential antiarrhythmic effects reported in our previous comprehensive review [5], experimental and clinical cardiologists retain interest in establishing their antiarrhythmic efficacy. A recent noteworthy study showed that β1-adrenoceptor autoantibody suppression is involved in the antiarrhythmic effects of omega-3 FA in male and female rats with essential hypertension [224]. The older rats in this strain exhibited lower omega-3 index than the normotensive controls [225]. Their antiarrhythmic effect was also demonstrated in the treatment of sympathetic over-drive in normotensive and hypertensive rats [226], and its supplementation in obese older female rats resulted in reduced heart susceptibility to VT and VF [227]. There was also concomitant reduction in plasma triglycerides, cholesterol and epicardial and retroperitoneal adipose tissue. The latter is known to promote cardiac arrhythmias, mainly due to the activation of pro-inflammatory signaling [228]. Most recent experimental findings revealed an antiarrhythmic effect of omega-3 FA supplementation in the condition of light pollution which is deleterious to the heart by promoting pro-inflammatory signaling [44]. In addition, the acute administration of EPA or DHA reduced the inducible VF in the adult male and female hereditary hypertriglyceridemic rats [192] and cardiac arrhythmias after cardiac infarction [222].

Although the majority of findings suggest omega-3 FA benefits [229], there is insufficient evidence for their routine implementation in clinical practice. A recent systematic review suggests that most studies found potential omega-3 FA benefit in coronary heart disease [230]. However, a more objective evaluation of its antiarrhythmic potential in clinical trials requires at least noting pre-intervention free blood plasma levels, but more appropriately, monitoring the red blood cell content expressed as omega-3 index. There was an important 1% increase in this index associated with 48% reduction in VF risk in patients suffering their first infarction [231]. A further ‘inadequacy’ was noted in clinical trials. This is high patient and treatment heterogeneity that could affect cohort analytic results. Finally, it is difficult to look for omega-3 FA antiarrhythmic effects in an optimal cardio-protective drug treatment regimen. As previously suggested, omega-3 FA antiarrhythmic efficacy strongly depends on the underlying clinical and pharmacological conditions [232], and intervention trials can become more effective by including a low omega-3 index in inclusion criteria. This would then create a study population more likely to identify omega-3 FA antiarrhythmic effects [233].

A previous review [5] recorded that omega-3 FA prevented occurrence of both AF and POAF (post-operative AF), and also occurrence of malignant ventricular arrhythmias in some cases. A recent randomized clinical trial involved healthy 50-year-and-over women and men who received omega-3 FA supplement during five years follow-up identified no effect on AF risk [234]. In contrast, supplementation terminated focal atrial tachycardia and failure of catheter ablation. This was considered most likely due to the effect on the autonomous nervous system [235]. Moreover, the alleviation of arrhythmic burden in children with frequent idiopathic premature ventricular contractions by omega-3 FA supplementation has also been reported [236,237]. This antiarrhythmic effect was most likely attributable to improved autonomic function, and this is consistent with previous findings in children with obesity. Additional reports include that the potential preventive role of omega-3 FA is associated with AF in hypertensive subjects [238]. Intra-operative infusion of omega-3 resulted in reduced POAF [239], and the J-MINUET sub-study showed that lower EPA/AA ratio is associated with fatal in-hospital arrhythmic events in those patients with acute myocardial infarction [240].

Contrasting research contends that omega-3 FA treatment can be undesirable, and even pro-inflammatory in some pathophysiological settings [241]. Inflammation-mediated specialized pro-resolving lipid mediators can be derived from arachidonic acid or omega-3 FA, and these can be released from the membrane in ischemia. Therefore, administration of omega-3 FA in acute myocardial infarction, ischemia and reperfusion [242] or sympathetic overdrive can be harmful, and even pro-arrhythmic [243]. Omega-3 FA alters cardiac electrophysiology and may be pro- or antiarrhythmic, depending on the arrhythmia mechanism, and acute interaction of the beta-adrenergic overdrive can be responsible for either survival or sudden death [226]. This partly explains the contradictory outcomes of clinical trials, and omega-3 FA should therefore be used with caution according to the specific conditions. According to VITAL research trial the supplementation with omega-3 FA did not result in a lower incidence of major cardiovascular events than placebo [244]. However, the association with plasma omega-3 index was missing. While according to recent systematic meta-analysis omega-3 FA reduced cardiovascular mortality and improved cardiovascular outcomes [245].

In summary, basal omega-3 FA-controlled studies under defined clinical indications are warranted to clearly establish the antiarrhythmic benefits. In addition, the current experimental findings must be verified and supported by molecular investigations. This will then provide greater objectivity in the evaluation of omega-3 FA antiarrhythmic efficacy, and this is essential for evidence-based medicine.

### Mechanisms Relevant to Antiarrhythmic Properties Omega-3 Fatty Acids

In concurrence with our previous review [5], available data suggests that the antiarrhythmic effects attributed to omega-3 FA include direct and indirect actions. These include modulation of ion channels and transporters’ properties, membrane composition and fluidity, anti-inflammatory and anti-fibrotic signaling and sympathetic-vagal balance. Free omega-3 FA from acute administration or release from the cardiac tissue affects ion channels and transporters and Ca^2+^ handling regulatory proteins. This directly results in acute myocardial electrophysiological alterations. In contrast, omega-3 FA incorporated in cell membranes from long-term supplementation indirectly alters cardiac electrical activity through changes in membrane properties [243,246]. 

Superfused omega-3-FA inhibits triggered arrhythmias in cardiomyocytes from rabbits and in patients with HF by lowering intracellular Ca^2+^ and reducing response to noradrenalin [247]. The omega-3 FA G protein-coupled-receptor targets modulate gene expression and transcription, lipoxidative processes and ROS release [222,248]. It is also important that omega-3 FA treatment inhibited IL-1β stimulated pro-arrhytmic loss of myocardial Cx43 protein and protection of Cx43 gap junction channel function by inhibiting NFκβ translocation [249]. This was associated with attenuated cardiac fibrosis following ischemic injury. It would therfore be beneficial to determine if omega-3 FA inhibits the CxHC mediated pro-inflammatory purinergic signaling which then activates pro-arrhythmic pro-fibrotic signaling [17]. Similarly, omega-3 FA up-regulated myocardial Cx43, attenuated its abnormal cardiomyocyte topology and the structural extracellular matrix remodeling in catecholamine overdrived normotensive and hypertensive rats [226]. This omega-3 FA treatment concurrently prevented increased the Cx43 variant phosphorylated at serine-368 and PKC which are known to modulate Cx43 distribution. These effects could underlie the omega-3 FA-increased threshold current required to induce VF in omega-3 FA treated rats. Finally, Cx43 upregulation and reduced propensity of the heart to inducible VF was demonstrated in the female obese rat model [227] and also in both male and female rats exposed to light pollution [44]. However, the omega-3 FA mechanisms affecting the expression and/or degradation of cardiac Cx43 and modulation of Cx43 channel function require elucidation. Cx43 is sensitive to redox status, and oxidative-nitrosative stress and Cx43 levels were blunted by administration of icosapentethyl which is a highly purified synthetic EPA derivative [222].

Omega-3 FA antiarrhythmic efficacy was further demonstrated when it was acutely administered in the perfused rat heart model [192]. This strongly indicates that the direct antiarrhythmic effect was most likely caused by modulating ion channels, and perhaps also Cx43 channel function and Ca^2+^ handling. The direct inhibition of Na^+^, Ca^2+^ and K^+^ sarcolemma ion channels in some pathological conditions could also stabilize electrical activity and prolong the cardiomyocyte relative refractory period [246,248,250,251,252,253]. In addition, the omega-3 FA incorporation prevented action potential shortening [254] and its modulation of intracellular Ca^2+^ handling [255], sarcoplasmic reticulum RyR channels inhibition and prevention of Ca^2+^ overload may abolish or attenuate arrhythmia triggers [256,257]. 

Omega-3 FA also modulated the L-type Ca^2+^ current, and could induce loss of the action potential dome [258], because the action potential was prolonged and Ito current densities were gradually reduced with increased DHA concentration [259]. Dietary omega-3 FA suppressed up-regulation of the Na^+^/H^+^-exchanger activity and lowered the incidence of delayed after-depolarization in the volume-and-pressure-overload rabbit model [260]. Further, DHA and EPA both blocked peak INa and reduced late INa [261], and omega-3 FA modulated IKs gating, channel expression and location in membrane micro-domains [262]. Noteworthy, omega-3 FA are involved in many mitochondrial processes. These include its Ca^2+^ homeostasis, gene expression, respiratory function, ROS production and apoptosis. The mitochondria therefore have an essential function in the protective effects of omgea-3 FA [263].

**Figure 4:**
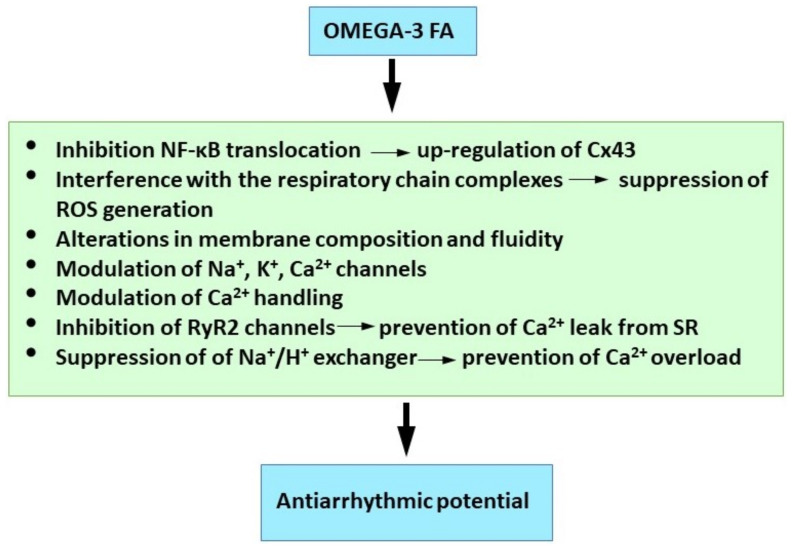
Molecular mechanisms of omega 3-FA underlying their antiarrhythmic properties. See references [5,246,249,256,260,263].

Taken together it appears that the beneficial and antiarrhythmic effects of the omega-3 FA are dependent on multiple synergistic action mechanisms (Figure 4) and the intimate association of their function and effect.

## 6. Conclusions

There is strong evidence from preclinical research and clinical studies that targeting inflammation and oxidative stress may provide a path to ameliorate cardiac arrhythmia burden. Indeed, cardioprotective SGLT2 inhibitors, statins and omega-3 FA exhibiting anti-arrhythmic properties are potent anti-oxidative and anti-inflammatory agents. These agents most likely affect the pro-arrhythmia primary mechanisms, such as triggered activity via modulation of Ca^2+^ handling and ions homeostasis as well as pro-fibrotic signaling likely by targeting CxHC and pannexin-1 channels. Consequently, it may result in protection of Cx43 gap junction channels mediated intercellular electrical coupling and communication. However, causal relationship is missing and this is the limitation of the studies included in this review. 

Therefore, further research should explore as whether SGLT2 inhibitors, statins and omega-3 FA as well as other cardio-protective compounds are able to control the NLRP inflammasome signaling via inhibition of CxHC and pannexin-1 channels. In this context it would be interesting to examine the efficacy of pharmacological blockade of CxHC and pannexin-1 channels, including specific inhibitors [17] (e.g., Gap19, Gap26, Gap27), to prevent development of cardiac arrhythmias. Moreover, comprehensive elucidation of downstream targets of anti-inflammatory compounds may provide further information about modulation of arrhythmia substrate. 

Nevertheless, evidence suggest that potent anti-inflammatory and anti-oxidative compounds impact pro-arrhythmic factors and the development of arrhythmia substrate. It may be a paradigm for the development of novel drugs aimed to prevent occurrence of life-threatening ventricular arrhythmias and deleterious atrial arrhythmias.

## Data Availability

Not applicable.
